# Pachychoroid neovasculopathy and age-related macular degeneration

**DOI:** 10.1038/srep16204

**Published:** 2015-11-06

**Authors:** Masahiro Miyake, Sotaro Ooto, Kenji Yamashiro, Ayako Takahashi, Munemitsu Yoshikawa, Yumiko Akagi-Kurashige, Naoko Ueda-Arakawa, Akio Oishi, Hideo Nakanishi, Hiroshi Tamura, Akitaka Tsujikawa, Nagahisa Yoshimura

**Affiliations:** 1 Department of Ophthalmology and Visual Sciences, Kyoto University Graduate School of Medicine, Kyoto, Japan; 2 Center for Genomic Medicine/Inserm U.852, Kyoto University Graduate School of Medicine, Kyoto, Japan; 3 Department of Ophthalmology, Kagawa University, Kagawa, Japan

## Abstract

Pachychoroid neovasculopathy is a recently proposed clinical entity of choroidal neovascularization (CNV). As it often masquerades as neovascular age-related macular degeneration (AMD), it is currently controversial whether pachychoroid neovasculopathy should be distinguished from neovascular AMD. This is because its characteristics have yet to be well described. To estimate the relative prevalence of pachychoroid neovasculopathy in comparison with neovascular AMD and to investigate the phenotypic/genetic differences of the two diseases, we evaluated 200 consecutive Japanese patients who agreed to participate in the genetic study and diagnosed with pachychoroid neovasculopathy or neovascular AMD. Pachychoroid neovasculopathy was observed in 39 individuals (19.5%), which corresponds to one fourth of neovascular AMD. Patients with pachychoroid neovasculopathy were significantly younger (*p* = 5.1 × 10^−5^) and showed a greater subfoveal choroidal thickness (*p* = 3.4 × 10^−14^). Their genetic susceptibility to AMD was significantly lower than that of neovascular AMD; *ARMS2* rs10490924 (*p* = 0.029), *CFH* rs800292 (*p* = 0.013) and genetic risk score calculated from 11 AMD susceptibility genes (*p* = 3.8 × 10^−3^). Current results implicate that the etiologies of the two conditions must be different. Thus, it will be necessary to distinguish these two conditions in future studies.

Age-related macular degeneration (AMD) is a major cause of progressive visual impairment in developed countries^1^,^2^,^3^. After the discovery of the major AMD susceptibility genes *ARMS2* and *CFH*^4^,^5^,^6^,^7^, AMD has been thought to be a genetically homogenous disease. However, it is well known that the clinical characteristics of neovascular AMD are somewhat different between Asians and Caucasians. For example, the prevalence of large soft drusen in the unaffected eye is significantly lower in Asians, compared with Caucasians^8^. Likewise, polypoidal choroidal neovascularization (PCV) is a common subtype of neovascular AMD in Asians, while this is not the case for Caucasians.^8^It seems unlikely that such a substantial heterogeneity of a single disease can be solely attributed to different ethnic characteristics.

It has been reported that patients with PCV often have a history of central serous chorioretinopathy (CSC). These two conditions share characteristics such as a thick choroid and a good response to photodynamic therapy. For these reasons, the association between PCV and CSC has been widely investigated^9^,^10^,^11^. Several groups have reported that eyes with neovascular AMD with choroidal vascular hyperpermeability, a characteristic of CSC, have a thicker choroid^12^,^13^. Furthermore, CNVs with choroidal hyperpermeability have a different genotype distribution from those without^14^. However, CNV in cases where there is a history of diagnosed CSC, or characteristics of CSC, is often misdiagnosed as a peculiar type of neovascular AMD. This applies even in cases where there are no drusen, since it had not been well established that CSC itself might lead to CNV.

Recently, several researchers have proposed a restructuring of the neovascular AMD, PCV, and CSC spectrum. In 2012, Fung *et al.* reported that type 1 CNV can develop in long standing CSC and can masquerade as neovascular AMD^15^. In 2013, Warrow *et al.* described a new clinical entity characterized by a range of retinal pigment epithelium (RPE) abnormalities overlying areas of choroidal thickening. The researchers termed the condition “pachychoroid pigment epitheliopathy (PPE)”^16^. In 2014, Pang and Freund reported a small series of cases involving patients with CNV occurring over areas of increased choroidal thickness and dilated choroidal vessels. They termed this “pachychoroid neovasculopathy”^17^. These eyes did not have drusen or degenerative changes to suggest AMD or other degenerative diseases. Pang and Freund speculated that the cause of pachychoroid neovasculopathy might be a pachychoroid-driven process such as choroidal congestion and choroidal hyperpermeability manifested by choroidal thickening and dilated choroidal vessels.

Pachychoroid neovasculopathy is characterized by thick choroid, RPE abnormalities, and/or choroidal vascular hyperpermeability. It has been suggested that it resides within a spectrum of diseases that includes PPE, CSC, and PCV. Most importantly, pachychoroid neovasculopathy often masquerades as neovascular AMD, so that the original report caution against the misdiagnosis of pachychoroid neovasculopathy as neovascular AMD^15^,^17^. We hypothesized that many patients with pachychoroid neovasculopathy may have been misdiagnosed in this way. This is particularly plausible in the case of Asian patients, in whom CSC is common. The purpose of this study was to estimate the relative prevalence of pachychoroid neovasculopathy compared with neovascular AMD using multiple imaging methods. Moreover, we investigated the differences between the two conditions with respect to clinical characteristics and genotype distribution.

## Results

A total of 200 patients met the criteria for the current study. The demographics of the study population are summarized in Table 1. Mean age was 74.3 yr (SD = 8.8 yr). Characteristics were not significantly different between right and left eyes. Pseudodrusen and calcified drusen were observed in 9.0% and 1.0–1.5% of the study population, respectively. Approximately 30% of the eyes had RPE abnormality independent of the CNV lesion, and 15% of the eyes had choroidal vascular hyperpermeability. Mean subfoveal choroidal thickness was 227 μm (SD = 101 μm) in right eyes and 229 μm (SD = 109 μm) in left eyes.

Thirty-nine (19.5%) of the participants were diagnosed with pachychoroid neovasculopathy, and 161 (80.5%) patients were diagnosed with neovascular AMD. The characteristics of both groups are shown in Table 2. Patients with pachychoroid neovasculopathy were significantly younger than those with neovascular AMD (68.7 yr, *vs*. 75.6 yr, respectively; *p* = 5.1 × 10^−5^). They also had greater subfoveal choroidal thickness (*p* = 3.4 × 10^−14^). Although RPE abnormality and choroidal vascular hyperpermeability were more common in patients with pachychoroid neovasculopathy (*p* = 7.9 × 10^−12^ and 4.5 × 10^−7^, respectively), they were also found in 30.4% and 13.6%, respectively, of patients with neovascular AMD. Although choroidal vascular dilatation and polypoidal lesion tended to exist more in pachychoroid neovasculopathy, the difference were not statistically significant (*p* = 0.093 and 0.11, respectively).

**Figure 1:**
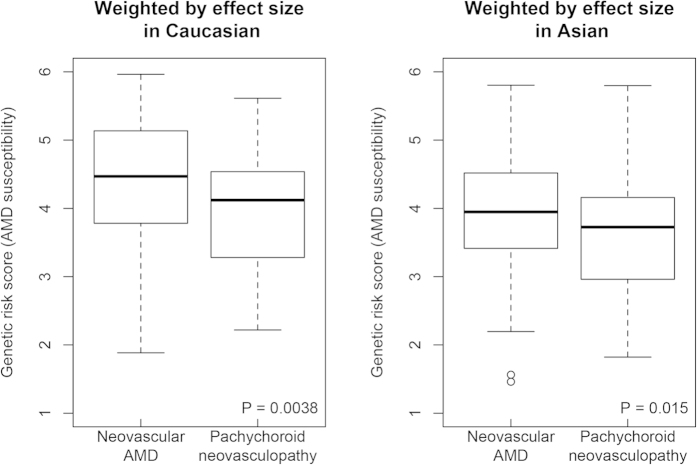
Distribution of genetic risk score calculated from 11 SNPs of 11 AMD susceptibility genes. We constructed a multi-locus genetic risk score by summing up the number of risk alleles of each single nucleotide polymorphism (SNP), weighted by its reported effect size (log odds ratio, OR). We evaluated both genetic risk score using effect size in Caucasian (**A**) and that using effect size in Asian (**B**). The effect sizes applied in this analysis are summarized in Supplementary Table 2. Pachychoroid neovasculopathy patients had significantly lower genetic risk scores for AMD than did neovascular AMD patients.

Table 3 and Fig. 1 show the genetic profiles of pachychoroid neovasculopathy and neovascular AMD. The genotype distribution of both *ARMS2* A69S and *CFH* I62V differed significantly between the two conditions (Table 3). The frequency of the T allele in *ARMS2* A69S was 51.3% and 64.8%, respectively, in patients with pachychoroid neovasculopathy and neovascular AMD (*p* = 0.029). The frequency of the A allele in *CFH* I62V was 41.0% and 25.5%, respectively, in patients with pachychoroid neovasculopathy and neovascular AMD (*p* = 0.013). These results showed that patients with pachychoroid neovasculopathy were less genetically susceptible to AMD.

Genetic risk score for AMD susceptibility also differed significantly between the pachychoroid neovasculopathy and neovascular AMD groups (OR = 0.52 and 0.56, respectively; *p* = 3.8 × 10^−3^ and 1.5 × 10^−2^; per one score increase of genetic risk score weighted by Asian, and per one score increase of genetic risk score weighted by Caucasian, respectively) (Fig. 1). Again, the pachychoroid neovasculopathy patients showed less genetic susceptibility to AMD.

**Figure 2:**
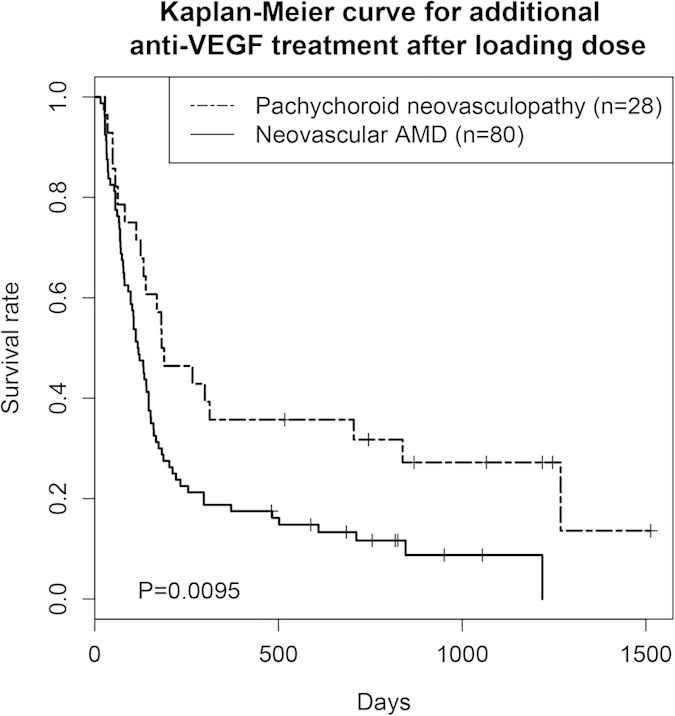
Kaplan-Meier curve for retreatment-free period after loading dose of ranibizumab. We selected study participants who had received a loading dose of ranibizumab (3 × one-monthly injections, 0.5 mg), been treated as needed afterwards, and been followed up for more than 12 months. The duration from the third ranibizumab injection to the administration of additional treatment (event), or to the final visit by June 2014, or loss to follow up) was reported on a daily basis. A log-rank test revealed that the curves for pachychoroid neovasculopathy and neovascular AMD differed significantly from one another *(p* = 0.0095). Pachychoroid neovasculopathy showed longer retreatment-free period.

A total of 108 patients treated with ranibizumab were eligible for the survival analysis. Of them, 28 individuals had pachychoroid neovasculopathy and 80 individuals had neovascular AMD (Table 4). The rate of dry macula after a loading dose did not differ significantly between the two groups (90.9% *vs*. 83.7%; *p* = 0.51). However, the Kaplan-Meier curves for the retreatment-free periods were significantly different between the two groups (*p* = 9.5 × 10^−3^). Pachychoroid neovasculopathy had a longer retreatment-free period (Fig. 2).

## Discussion

Pachychoroid neovasculopathy is a new clinical entity of CNV, which is characterized by its shared features with CSC or PPE^17^. However, pachychoroid neovasculopathy often masquerades as neovascular AMD, and standard diagnostic criteria have not yet been established. Since pachychoroid neovasculopathy may respond differently to photodynamic therapy or anti-VEGF therapy, discriminating it from neovascular AMD is of great importance. We hypothesized that pachychoroid neovasculopathy may commonly have been misdiagnosed as AMD, especially in Asians, and that this may partly explain the heterogeneity seen in neovascular AMD between Asians and Caucasians^8^. To estimate the prevalence of pachychoroid neovasculopathy, we investigated 200 CNV patients aged over 50, classifying them into pachychoroid neovasculopathy and neovascular AMD groups based on their phenotypes. In our cohort, pachychoroid neovasculopathy was seen with high frequency (one-fourth of neovascular AMD-diagnosed patients). In addition, our study revealed significantly different genetic backgrounds between the two groups.

As Table 1 shows, the overall clinical characteristics of the participants are similar to those previously reported in Japanese AMD patients. For example, pseudodrusen were observed in 9.0% of the patients, which is comparable to previous reports showing the prevalence of pseudodrusen in Japanese patients with AMD (10.8–16.8%)^18^,^19^. Our mean subfoveal choroidal thickness (227 ± 101 μm in right eyes and 229 ± 109 μm in left eyes) was also similar to that found in previous reports (subfoveal choroidal thickness; 204–245 μm in neovascular AMD and 243–293 μm in PCV)^12^,^20^. Considering the prevalence of choroidal vascular hyperpermeability in cases of PCV has been reported to be 9.8–34.8%^11^,^12^,^13^, the prevalence of choroidal vascular hyperpermeability in the current study (16.5% in right eyes and 14.0% in left eyes) is comparable to that in previous reports.

Based on our diagnostic criteria, 39 patients (19.5%; confidence interval [CI], 14.0%–25.0%) were diagnosed with pachychoroid neovasculopathy. Patients with pachychoroid neovasculopathy were significantly younger than those with neovascular AMD, which was compatible with the original report^17^. In the current study, however, we evaluated only patients with age of more than 50 years because the main interest of this study was to contrast pachychoroid neovasculopathy to neovascular AMD. Thus, mean age of the patients with pachychoroid neovasculopathy could be rather younger. Since pachychoroid neovasculopathy, unlike neovascular AMD, is characterized by a lack of drusen, and the genetic backgrounds differed significantly between the two groups (Table 3, Fig. 1), the etiologies of these two conditions are likely to be different.

As shown in Table 3, the *ARMS2* rs10490924 effect size for neovascular AMD (OR = 3.17) was higher than the previously reported effect size for Japanese neovascular AMD (OR = 2.41)^21^, while it was similar to the same effect size for Caucasian neovascular AMD (OR = 3.67)^22^. It is possible that pachychoroid neovasculopathy was misdiagnosed and included among neovascular AMD cases in previous reports. This would have led to an underestimation of the effect of AMD susceptibility genes in Japanese patients. Furthermore, a similar underdiagnosis of pachychoroid neovasculopathy may have occurred in other Asian studies, considering the higher prevalence of CSC in Asia^23^,^24^. Indeed, in *The AMD Gene Consortium* paper, the effect sizes of *ARMS2* rs10490924 and *CFH* rs10737680 for AMD were higher in Caucasian than in Asian patients, despite a lower prevalence of geographic atrophy, which is associated with a lower risk allele frequency, in Asians (Supplementary Figures 2A and 2B of *The AMD Gene Consortium* paper)^25^. Interestingly, the *CFH* rs800292 A allele frequency in pachychoroid neovasculopathy patients was comparable to that in normal Japanese subjects (41.0% vs. 40.5%, *p* = 0.92). Furthermore, these genetic findings were quite similar to our previous report that compared consecutive CNVs with choroidal vascular hyperpermeability to those without^14^, CNVs with choroidal vascular hyperpermeability, which would correspond to pachychoroid neovasculopathy at least in part, had normal genotype distribution in terms of *ARMS2* rs10490924 and *CFH* rs800292. Considering above, these CNVs should be distinguished from neovascular AMD.

Freund and colleagues hypothesized that pachychoroid neovasculopathy is associated with PCV^16^,^17^. Consistent with this, 56.4% of pachychoroid neovasculopathy cases are associated with polypoidal lesion. It follows that PCV figures may have been more augmented by misdiagnosed pachychoroid neovasculopathy cases than the figures of other subtypes. This may explain why PCV have thicker choroid^20^,^26^, more frequent history of CSC^11^,^27^,^28^, and more favorable prognosis^29^, particularly in the Asian population. The results may also explain why the effect of *ARMS2* rs10490924 is higher for AMD than for PCV in the Asian population^30^,^31^. All that being said, in our study 42.9% of eyes with neovascular AMD also had polypoidal lesion. Polypoidal legion is increasingly thought to be a manifestation of long-standing type 1 CNV in AMD as well as a variety of other diseases. In general, PCV has been considered as a subtype of neovascular AMD that accompanies polypoidal legion; thus, we suggest that pachychoroid neovasculopathy with polypoidal lesion should be distinguished from neovascular AMD with polypoidal lesion so as not to confuse the concepts of neovascular AMD and pachychoroid neovasculopathy.

To distinguish neovascular AMD and pachychoroid neovasculopathy is important not only because it is related to the etiology of CNV, but also because it can influence disease management. Our survival analysis showed that patients with pachychoroid neovasculopathy had a significantly longer retreatment-free period than those with neovascular AMD (*p* = 9.5 × 10^−3^) after a loading dose of anti-VEGF therapy. This indicates that pachychoroid neovasculopathy may be more self-limited or that VEGF secretion in pachychoroid neovasculopathy may be lower than in neovascular AMD. This hypothesis is compatible with the following observations: (1) CNV development takes a long time in pachychoroid neovasculopathy; and (2) the VEGF concentration in the aqueous humor of PCV eyes, whose figures may have been more bloated by misdiagnosed pachychoroid neovasculopathy, was reported to be lower than that of neovascular AMD eyes^32^. On the other hand, the dry macula rate after loading dose was over 80% in both conditions, revealing no statistically significant difference between them (*p* = 0.51). That said, it must be stated that the loading dose may have been adequate to eliminate VEGF in both conditions except for 10%–20% of patients, who may have been non-responder.

While the current study has significant implications, it does have limitations. Firstly, the current criteria for diagnosing pachychoroid neovasculopathy may not be ideal. Based on previous reports, we set our original diagnostic criteria – choroidal thickness, absence of drusen, choroidal vascular hyperpermeability, and RPE abnormalities – because no standard diagnostic criteria for pachychoroid neovasculopathy have been established so far. The current criteria could be more sophisticated to distinguish pachychoroid neovasculopathy and neovascular AMD with higher sensitivity and specificity. For instance, choroidal thickness varies with age and axial length^33^, so these parameters could be taken into account for diagnosis. As another example, we did not diagnose pachychoroid neovasculopathy when there was only one soft drusen in the fellow eye, in order to increase the specificity of the diagnosis. Ideally, the scoring method could have been employed to balance the sensitivity and specificity of the pachychoroid neovasculopathy diagnosis. Nonetheless, even though our criteria were based solely on phenotype, they successfully demarcated a cluster of CNV that was genetically distinct from neovascular AMD. Secondly, this study only included Japanese individuals. Considering the higher prevalence of CSC in Asians than in Caucasians, we speculate that pachychoroid neovasculopathy may have been misclassified as neovascular AMD in Asians more often. Future studies including other ethnicities are indicated. Thirdly, the significant result in the survival analysis may need to be deducted from the study because it is based on retrospective data.

In conclusion, pachychoroid neovasculopathy was different from neovascular AMD not only phenotypically but also genetically. Pachychoroid neovasculopathy may represent up to one quarter of diagnosed neovascular AMD cases. Although pachychoroid neovasculopathy often masquerades as neovascular AMD, their etiology is likely to be different because pachychoroid neovasculopathy shows lack of drusen and the genotype distribution of AMD susceptibility SNPs differed significantly between the two conditions. Pachychoroid neovasculopathy should be distinguished from neovascular AMD in future epidemiological and genetic studies. Lastly, further research is necessary to manage and prevent this disease.

## Methods

The current study was approved by the Institutional Review Board at Kyoto University Graduate School of Medicine and adhered to the tenets of the Declaration of Helsinki. Written informed consent was obtained from each genotyped patient.

### Subjects

We retrospectively reviewed the medical records of consecutive patients (1) who had visited the macular service of Kyoto University Hospital (Kyoto, Japan) between January 2010 and October 2012, agreed to participate in the genetic study (the participation rate ≥99%), and were genome-scanned, (2) who were diagnosed with either pachychoroid neovasculopathy or neovascular AMD (diagnostic criteria of these diseases are described in the following section), and (3) who were older than 50 years. Patients with the following conditions were excluded from the study: (1) CNV secondary to high myopia (spherical equivalent, ≤ − 6.00 D or axial length of ≥26 mm), trauma, angioid streaks, uveitis, or any other neovascular maculopathy, (2) choroidal thickness not available due to thick hemorrhage, (3) history of ocular surgery other than cataract surgery. Smoking status was evaluated using the Brinkman Index ([number of cigarettes per day] × [number of years smoking])^34^.

### Multimodal Imaging Methods

All patients underwent a complete ophthalmologic examination, including measurement of best-corrected visual acuity, determination of intraocular pressure, indirect ophthalmoscopy, slit-lamp biomicroscopy with a non-contact lens, color fundus photography, infrared reflectance (IR), fundus autofluorescence (FAF), fluorescein angiography (FA), indocyanine green angiography (IA), and spectral-domain optical coherence tomography (SD-OCT).

Color fundus photographs (field, 40°) were obtained digitally using a Topcon TRC NW6S non-mydriatic retinal camera (Topcon, Tokyo, Japan) after medical dilatation of the pupil (phenylephrine 0.5% and tropicamide 0.5%). IR, FAF, FA, and IA images were acquired using a confocal SLO (Spectralis HRA+OCT; Heidelberg Engineering, Heidelberg, Germany). IR images were obtained using a light stimulus of 820 nm. FAF images were obtained using an excitation light of 488 nm and a barrier filter beginning at 500 nm. The field of view was set to 30° × 30° centered on the macula. SD-OCT was conducted using a Spectralis HRA+OCT (Heidelberg Engineering). First, horizontal and vertical line scans through the fovea center were obtained at a 30° angle, followed by serial horizontal scans with an examination field size of 30° × 10°. Inverted OCT images, which enable us to measure choroidal thickness, were routinely obtained in all patients using an enhanced-depth imaging (EDI) technique^35^. At each location of interest on the retina, fifty SD-OCT images were acquired and averaged to reduce speckle noise.

### Image Analysis and Phenotyping

Soft drusen were graded based on the fundus photographs according to the simplified severity scale for AMD from the Age-Related Eye Disease Study (AREDS)^36^. The occurrence of dot- or ribbon-type pseudodrusen was confirmed using color fundus photography, IR, FAF, IA (late phase), or when there was OCT evidence of definite drusenoid deposits above the RPE.

**Figure 3:**
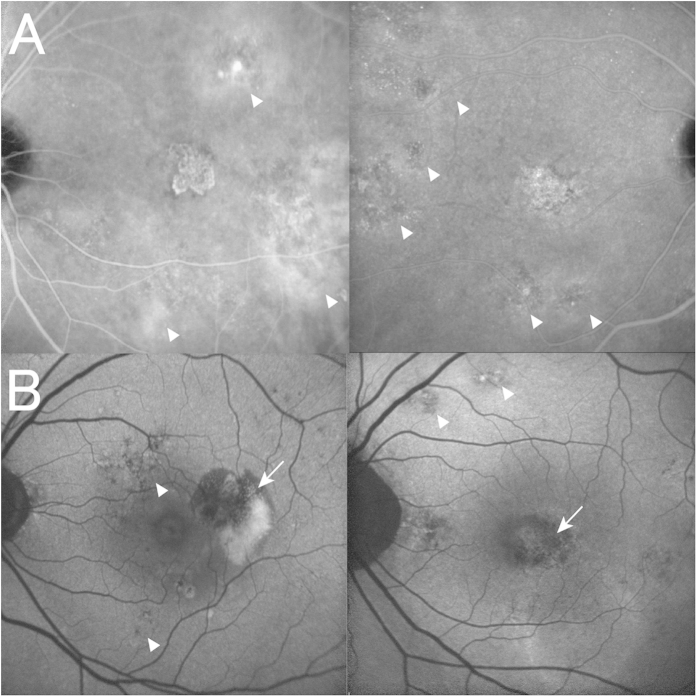
(**A**) Choroidal vascular hyperpermeability. Choroidal vascular hyperpermeability is evidenced by focal areas of hyperfluorescence, which appear during the middle phase of indocyanine green angiography (IA) and expand over time, eventually forming a ring shape (arrowheads). The center of the initially hyperfluorescent area becomes hypofluorescent during the late phase. (**B**) Retinal pigment epithelium abnormality. Retinal pigment epithelium (RPE) abnormality is seen as patchy areas of granular hypoautofluorescence with occasional discrete hyperautofluorescent specks scattered throughout the fundus autofluorescence (FAF) images^16^,^17^ (arrowheads). However, this was not considered indicative of RPE abnormality when this finding was adjacent to a choroidal neovascularization (CNV; arrows) lesion.

Subfoveal choroidal thickness and choroidal vascular hyperpermeability were evaluated as previously described^14^. Briefly, subfoveal choroidal thickness was defined as the vertical distance between Bruch’s membrane and the chorioscleral interface at the fovea, which was manually measured in the EDI-OCT images by a retinal specialist blinded to study parameters using the built-in caliber. Choroidal vascular hyperpermeability was determined by detecting multifocal hyperfluorescent areas with blurred margin that expanded during the late phase of IA (*i.e.*, 10–15 minutes after dye injection) (Fig. 3A). Choroidal vascular hyperpermeability was confirmed only when independent judgments of two retinal specialists (M.M. and S.O.) were agreed. The number of quadrants with dilated choroidal vessels was also evaluated for each eye, based on wide-field IA images taken five to ten minutes after dye injection.

RPE abnormality was determined by detecting patchy areas of granular hypoautofluorescence with occasional discrete hyperautofluorescent specks scattered throughout the FAF image, according to the original report of PPE^16^. RPE abnormality is often seen in CNV legion; therefore, only RPE abnormalities occurring independent of CNV lesions were evaluated in this study (Fig. 3B). The presence of RPE abnormality was only confirmed when independent judgments of two retinal specialists (M.M. and S.O.) were agreed.

### Definition of pachychoroid neovasculopathy

**Figure 4:**
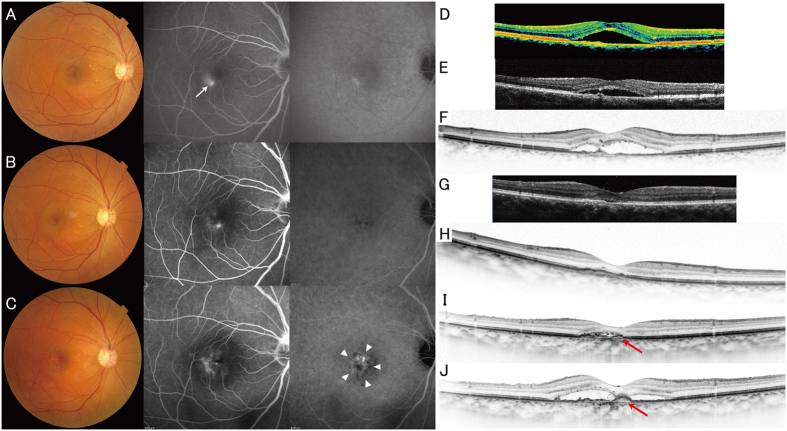
A case of pachychoroid neovasculopathy. A 50-year-old man visited the macular service at Kyoto University Hospital with a chief complaint of central scotoma in his right eye. (**A,D**) The color fundus photograph from the patient’s first visit shows serous retinal detachment without drusen. Fluorescein angiography (FA) shows late leakage (ink blot pattern; white arrow). Indocyanine green angiography (IA) image shows no choroidal neovascularization (CNV). Optical coherence tomography (OCT; vertical scan thorough the center of the fovea) shows serous retinal detachment and an almost flat retinal pigment epithelium (RPE) band. All these findings were compatible with a diagnosis of central serous chorioretinopathy (CSC). (**E**) Four months after the patient’s first visit: OCT shows persistent serous retinal detachment. (**B,F**) Ten months after the patient’s first visit: CNV is not apparent in either FA or IA. However, there are several protrusions in the OCT image. (**G–I**) 2.5 yr (**G**), 4.5 yr (**H**), and 6.5 yr (**I**) after the patient’s first visit. After spontaneous resolution of subretinal fluid, it again increased. The RPE band gradually elevated, suggesting the development of CNV (red arrows). (**C,J**) Seven years after the patient’s first visit, CNV is apparent in both FA/IA (white arrowheads) and OCT (red arrow) images. Choroid is thick all over the macula. Color fundus photography contains no drusen.

**Figure 5:**
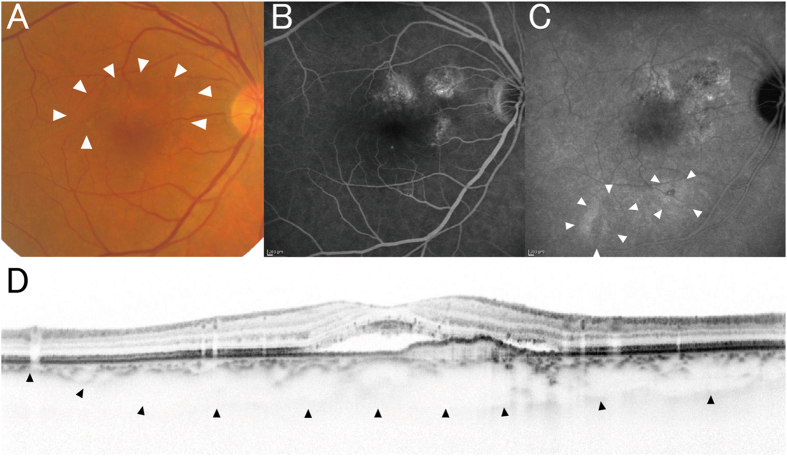
A case of pachychoroid neovasculopathy. A 68-year-old male was visually impaired in the right eye (best-corrected visual acuity = 30/20). (**A**) Color fundus photograph shows serous retinal detachment (arrowheads) without drusen. (**B**) FA suggests occult CNV. (**C**) Late-phase IA shows choroidal vascular hyperpermeability (arrowheads). (**D**) Enhanced depth imaging OCT (vertical scan thorough the center of the fovea) reveals type 1 CNV and subretinal fluid. Choroid is thick throughout the macula, and subfoveal choroidal thickness was measured as 353 μm. He had GT genotype at *ARMS2* A69S (rs10490924) and AG genotype at *CFH* I62V (rs800292).

**Figure 6:**
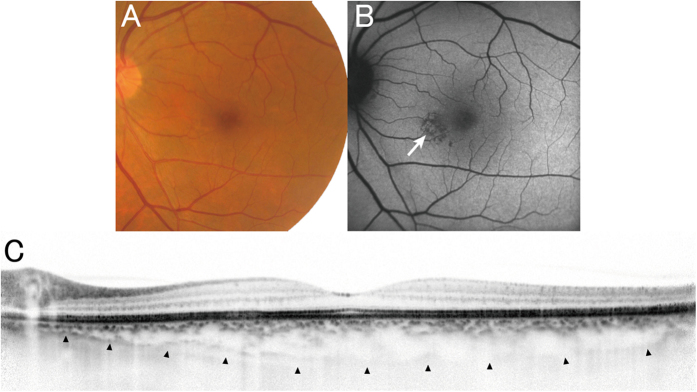
Pachychoroid pigment epitheliopathy observed in the fellow eye of the patient in Fig. 5. **(A)** Color fundus photography shows no drusen. (**B)** FAF shows RPE abnormality associated with choroidal vascular dilation (arrow), but without hyperautofluorescent lesions, which would suggest the previous presence of subretinal fluid. (**C**) Enhanced depth imaging OCT (horizontal scan thorough the center of the fovea) shows thick choroid throughout the macula, and subfoveal choroidal thickness was 335 μm. All these findings are compatible with a diagnosis of pachychoroid pigment epitheliopathy.

Although there is no established definition for pachychoroid neovasculopathy, the nature of this entity is type 1 CNV secondary to CSC or PPE, which often masquerades as AMD^15^,^17^. In this study, pachychoroid neovasculopathy was diagnosed if all of the following criteria were met: (1) CNV in either eye; (2) subfoveal choroidal thickness ≥200 μm in both eyes; (3) no drusen or only non-extensive (total area, ≤125 μm circle) hard drusen (≤63 μm) in both eyes (AREDS category 1, no AMD); (4) CSC or PPE characteristics; namely, choroidal vascular hyperpermeability (Fig. 3A), RPE abnormality independent of CNV lesion (Fig. 3B), the presence of dilated choroidal vessels or thickening below the type 1 CNV, or a history of CSC. Cases identified pachychoroid neovasculopathy are shown in Figs 4, 5, 6. Patients with CNV and other findings corresponding to AREDS categories 2, 3, and 4 (extensive hard drusen, soft drusen [intermediate, ≥63 and <125 μm; large, ≥125 μm], pseudodrusen, focal hyperpigmentation, or geographic atrophy) were diagnosed with neovascular AMD.

Choroidal thickness is an important factor that characterizes PPE, though it is not included in the definition of CSC. Although the original report did note “focal, multifocal, or diffuse area of reddish orange background within the arcades with minimal to absent choroidal vascular markings (reduced fundus tessellation), indicative of a thickened choroid,” it was somewhat subjective^16^. For this reason, we employed a choroidal thickness threshold value instead. Because the original report presented a PPE case with a subfoveal choroidal thickness of 231 μm, we set this threshold value at 200 μm. In another study, the mean subfoveal choroidal thickness in CSC eyes was 414 μm (95% range, 200 μm–628 μm), while that of the contralateral eyes was 350 μm (95% range, 123–577 μm)^37^. This further supports the validity of our threshold value.

### Genotyping

Genotyping was performed using the Illumina BeadChip; HumanOmni2.5, OmniExpress and/or HumanExome. Quality controls were conducted using PLINK ver1.07 (http://pngu.mgh.harvard.edu/~purcell/plink/), and missing values were imputed using SHAPEIT2 (http://www.shapeit.fr/) and IMPUTE2 (https://mathgen.stats.ox.ac.uk/impute/impute_v2.html) software. Details are described in Supplementary Notes.

As a reference group, we used 3,248 unrelated individuals from the general population, recruited from the Nagahama Prospective Genome Cohort for the Comprehensive Human Bioscience (The Nagahama Study)^38^,^39^,^40^. These patients were genotyped using HumanHap610 K quad arrays, HumanOmni2.5 M arrays, and/or HumanExome arrays (Illumina Inc., San Diego, CA). The two SNP genotypes were extracted from the cohort’s fixed dataset.

### Gene/SNP selection and genetic risk score

From the 19 genes that were significantly associated with AMD in the report by *The AMD Gene Consortium*^25^, we used 11 genes whose associations with AMD were also verified in Asian individuals (*Gene for AMD in Asian [GAMA] consortium*)^41^. For *CFH*, *C3*, and *CETP*, we investigated single nucleotide polymorphisms (SNPs) that have commonly been reported in Asians^14^,^38^,^42^,^43^,^44^,^45^, rather than those reported by *The AMD Gene Consortium*. Altogether, the following SNPs were investigated in this study: *ARMS2* (rs10490924), *CFH* (rs800292), *C2/CFB* (rs429608), *C3* (rs2241394), *APOE* (rs4420638), *CETP* (rs3764261), *VEGFA* (rs943080), *TNFRSF10A* (rs13278062), *CFI* (rs4698775), *TGFBR1* (rs334353), and *ADAMTS9* (rs6795735).

We constructed a multi-locus genetic risk score by summing up the number of risk alleles of each SNP, weighted by their reported effect sizes (log odds ratio, OR). Because of the possibility that the effect size in Asians did not reflect the true AMD susceptibility because of errors in the sample introduced by wrongly diagnosed cases of pachychoroid neovasculopathy, we evaluated genetic risk score using the effect sizes in both Caucasian and Asian populations. The effect sizes applied in this analysis are summarized in Supplementary Table 2.

### Survival analysis

To evaluate the clinical importance of pachychoroid neovasculopathy, we selected study participants who had received a loading dose of ranibizumab (3 monthly injections, 0.5 mg), received treatment as needed afterwards, and been followed up for more than 12 months. After excluding the patients who had had previous ocular treatment (other than cataract surgery), and those with a visual acuity less than 20/200, we retrospectively reviewed medical charts. The durations from their third ranibizumab injection to event (the administration of additional treatment) or censoring (final visit by June 2014, or loss to follow up) was reported on a daily basis.

### Statistical analysis

Every 2 × 2 table was evaluated using Fisher’s exact test. Continuous variables were compared using the unpaired t-test. Genotype distribution was compared using the chi-square test for trends. Survival analysis on the retreatment-free period was conducted using the Kaplan-Meier method. Differences between curves were evaluated using the log-rank test. A *p*-value < 0.05 was considered statistically significant. These statistical analyses were conducted using R software ver.3.02 (http://www.r-project.org/). For the survival analysis, the CRAN package “survival” was employed.

## Additional Information

**How to cite this article**: Miyake, M. *et al.* Pachychoroid neovasculopathy and age-related macular degeneration. *Sci. Rep.* **5**, 16204; doi: 10.1038/srep16204 (2015).

## Supplementary Material

Supplementary Information

## Figures and Tables

**Table 1:** Characteristics of the study population.

	Laterality	
	Right eyes	Left eyes
Number of patients	200	
Age (years)	74.3 ± 8.8‡	
Sex (male:female)	143:57	
Refraction (diopter)	0.24 ± 2.30‡	0.23 ± 2.19‡
Drusen		
No drusen or only non-extensive hard drusen	41 (20.5%)	42 (21.0%)
Large soft drusen	52 (26.0%)	53 (26.5%)
Extensive soft drusen*	33 (16.5%)	27 (13.5%)
Pseudodrusen	18 (9.0%)	18 (9.0%)
Calcified drusen	3 (1.5%)	2 (1.0%)
RPE abnormality apart from the CNV legion	61 (30.5%)	58 (29.0%)
Choroidal vascular dilatation (0:1:2:3)†	123:56:20:1	134:48:14:4
Choroidal vascular hyperpermeability	33 (16.5%)	28 (14.0%)
Subfoveal choroidal thickness (μm)	227± 101‡	229 ± 109‡

^*^More than 6 drusen (>63 μm each) in ETDRS grid.

^†^Number of quadrants.

^‡^mean ± standard deviation are shown.

AREDS: Age-related Eye Disease Study, RPE: retianl pigment epithelium. CNV: choroidal neovascularization. ETDRS: early treatment diabetic retinopathy study.

**Table 2:** Characteristics of the pachychoroid neovasculopathy and neovascular AMD.

	Pachychoroid neovasculopathy	Neovascular AMD	*P*-value
Number of patients	39 (19.5%)	161 (80.5%)	–
Age (years)	68.7 ± 9.0	75.6 ± 8.3	5.1 × 10^−5^
Sex (male:female)	30:9	113:48	0.44
Refraction (diopter)*	0.57 ± 2.08	0.10 ± 2.17	0.22
RPE abnormality apart from the CNV legion	35 (89.7%)	49 (30.4%)	7.9 × 10^−12^
Chorodial Vessels dilatation (number of quadrants)	0.64 ± 0.73	0.43 ± 0.58	0.093
Choroidal vascular hyperpermeability	21 (53.8%)	22 (13.6%)	4.5 × 10^−7^
Subfoveal choroidal thickness (μm)	310`± 53	208 ± 100	3.4 × 10^−14^
Polypoidal legion	22 (56.4%)	69 (42.9%)	0.11
Brinkman Index	482 ± 495	537 ± 625	0.56

AMD: age-related macular degeneration, RPE: retinal pigment epithelium.

CNV: choroidal neovascularization.

^*^Phakic eyes only; Average of both eyes are presented if both eyes are phakic.

**Table 3:** Genotypic differences between the patients with pachychoroid neovasculopathy and neovascular AMD.

	Data from	n	ARMS2 A69S (rs10490924)						CFH I62V (rs800292)				
			GG	GT	TT	>T-allele frequency	P*	Odds ratio (vs control)	AA	AG	GG	A-allele frequency	P*
Normal (Japanese)	Nagahama study	3,248	1312	1499	435	36.5%	–	–	546	1538	1162	40.5%	−
Pachychoroid neovasculopathy	Current study	39	11	16	12	51.3%	**0.029**	1.83†	8	16	15	41.0%	**0.013**
Neovascular AMD	Current study	161	24	64	71	64.8%		3.20†	17	48	96	25.5%	
Neovascular AMD (Japanese)	Arakawa *et al.*	1,536	–	–	–	57.4%	–	2.41‡	–	–	–	27.1%	–
Neovascular AMD (Caucasian)	Sobrin *et al.*	1,775	–	–	–	–	–	3.67‡	–	–	–	–	–

^*^Adjusting for age and sex.

^†^Compared to 3,248 normal Japanese.

^‡^Cited from the original paper.

CSC: central serous chorioretinopathy, AMD: age-related macular degeneration, CNV: choroidal neovascularization.

**Table 4:** Summary of the patients who were eligible for the survival analysis.

	Pachychoroid neovasculopathy	Neovascular AMD
Number of patients	22	86
Age (years)	68.7 ± 8.5	74.1 ± 8.2
Sex (male:female)	5:17	27:59
Choroidal vascular hyperpermeability	15 (68.2%)	15 (17.4%)
Subfoveal choroidal thickness (μm)	314 ± 58	227 ± 103
Polypoidal legion	11 (50.0%)	41 (47.7%)
Dry macula after loading dose	20 (90.9%)	72 (83.7%)

AMD: age-related macular degeneration.
