# Supplementary material for: Pachychoroid neovasculopathy and age-related macular degeneration
Source: Sci Rep. 2015 Nov 6;5:16204. doi: 10.1038/srep16204 (PMC4635432; doi:10.1038/srep16204)
Supplement: Supplementary Information [file srep16204-s1.doc]

**Supplementary Information**

**Pachychoroid neovasculopathy and age-related macular degeneration**

Masahiro Miyake, MD^1,2^, Sotaro Ooto, MD, PhD^1^, Kenji Yamashiro, MD, PhD^1^, Ayako Takahashi, MD^1^, Munemitsu Yoshikawa, MD^1^, Yumiko Akagi-Kurashige, MD^1^, Naoko Ueda-Arakawa MD^1^, Akio Oishi, MD, PhD^1^, Hideo Nakanishi, MD, PhD^1^, Hiroshi Tamura, MD, PhD, ScM^1^, Akitaka Tsujikawa, MD, PhD^1,3^, Nagahisa Yoshimura, MD, PhD^1^

^1^Department of Ophthalmology and Visual Sciences, Kyoto University Graduate School of Medicine, Kyoto, Japan.

^2^Center for Genomic Medicine/Inserm U.852, Kyoto University Graduate School of Medicine, Kyoto, Japan

^3^Department of Ophthalmology, Kagawa University, Kagawa, Japan

**Supplementary Materials**

**Supplementary Table 1**

| Supplementary Table 1. Imputation quality | | | |  |  |  |  |  |  |
| --- | --- | --- | --- | --- | --- | --- | --- | --- | --- |
| Gene | SNP | Chr | Position | Effect allele^*^ | EAF | Impute status | Info value^†^ | Certainty value^†^ | EAF in directly-genotyped samples |
| ARMS2 | rs10490924 | 10 | 124214448 | T | 0.578 | - | - | - | - |
| CFH | rs800292 | 1 | 196642233 | G | 0.739 | - | - | - | - |
| C2/CFB | rs429608 | 6 | 31930462 | G | 0.938 | - | - | - | - |
| C3 | rs2241394 | 19 | 6685230 | C | 0.074 | Partly imputed | 0.853 | 0.968 | 0.062 |
| APOE | rs4420638 | 19 | 45422946 | T | 0.929 | - | - | - | - |
| CETP | rs3764261 | 16 | 56993324 | A | 0.224 | - | - | - | - |
| VEGFA | rs943080 | 6 | 43826627 | T | 0.330 | - | - | - | - |
| TNFRSF10A | rs13278062 | 8 | 23082971 | T | 0.396 | Partly imputed | 0.964 | 0.977 | 0.396 |
| CFI | rs4698775 | 4 | 110590479 | G | 0.240 | Partly imputed | 0.995 | 0.997 | 0.221 |
| TGFBR1 | rs334353 | 9 | 101908365 | T | 0.499 | Partly imputed | 0.999 | 0.999 | 0.500 |
| ADAMTS9 | rs6795735 | 3 | 64705365 | T | 0.869 | Partly imputed | 0.995 | 0.998 | 0.873 |
| * Risk allele for AMD development. | | |  |  |  |  |  |  |  |
| † These data are provided by IMPUTE2 as quality value. | | | | |  |  |  |  |  |

**Supplementary Table 2**

| Supplementary Table 2. Reported effect size for the susceptibility of AMD. | | | | | | |  |  |
| --- | --- | --- | --- | --- | --- | --- | --- | --- |
| Gene | SNP | Chr | Position | Effect allele^*^ | Asian | | Caucasian | |
|  |  |  |  |  | Odds ratio | Weight^†^ | Odds ratio | Weight^†^ |
| ARMS2 | rs10490924 | 10 | 124214448 | T | 2.42 | 0.384 | 2.76 | 0.441 |
| CFH | rs800292 | 1 | 196642233 | G | 1.69 | 0.229 | 1.73^c^ | 0.238 |
| C2/CFB | rs429608 | 6 | 31930462 | G | 1.35 | 0.131 | 1.74 | 0.241 |
| C3 | rs2241394 | 19 | 6685230 | G | 2.08^a^ | 0.319 | 1.42^‡^ | 0.152 |
| APOE | rs4420638 | 19 | 45422946 | T | 1.16 | 0.066 | 1.30 | 0.114 |
| CETP | rs3764261 | 16 | 56993324 | A | 1.41 | 0.149 | 1.15^d^ | 0.061 |
| VEGFA | rs943080 | 6 | 43826627 | T | 1.10 | 0.041 | 1.15 | 0.061 |
| TNFRSF10A | rs13278062 | 8 | 23082971 | T | 1.37^b^ | 0.137 | 1.15 | 0.061 |
| CFI | rs4698775 | 4 | 110590479 | G | 1.17 | 0.068 | 1.14 | 0.057 |
| TGFBR1 | rs334353 | 9 | 101908365 | T | 1.10 | 0.041 | 1.13 | 0.053 |
| ADAMTS9 | rs6795735 | 3 | 64705365 | T | 1.23 | 0.092 | 1.10 | 0.041 |
| * Risk allele for the AMD development, † Logarithm of odds ratio, ‡ Odds ratio of C3/rs2230199 is applied. | | | | | | | | |
| All data came from “*The AMD Gene Consortium*”[^1^](#_ENREF_1) or “*Genetics for AMD in Asian consortium*”[^2^](#_ENREF_2) except for the followings; | | | | | | | | |
| a: Data from reference 3[^3^](#_ENREF_3) (Japanese dataset), b: Data from reference 4[^4^](#_ENREF_4) (Japanese dataset) | | | | | | | | |
| c: Data from reference 5[^5^](#_ENREF_5) (Caucasian meta-analysis), d: Data from reference 6[^6^](#_ENREF_6). | | | | | | | | |

**References for Supplementary Table 2**

1. Fritsche LG, Chen W, Schu M, et al. Seven new loci associated with age-related macular degeneration. Nat Genet 2013;45:433-9, 9e1-2.

2. Cheng CY, Yamashiro K, Jia Chen L, et al. New loci and coding variants confer risk for age-related macular degeneration in East Asians. Nature Communications 2015;6:6063.

3. Yanagisawa S, Kondo N, Miki A, et al. A common complement C3 variant is associated with protection against wet age-related macular degeneration in a Japanese population. PloS one 2011;6:e28847.

4. Arakawa S, Takahashi A, Ashikawa K, et al. Genome-wide association study identifies two susceptibility loci for exudative age-related macular degeneration in the Japanese population. Nat Genet 2011;43:1001-4.

5. Yuan D, Yang Q, Liu X, et al. Complement factor H Val62Ile variant and risk of age-related macular degeneration: a meta-analysis. Mol Vis 2013;19:374-83.

6. Yu Y, Bhangale TR, Fagerness J, et al. Common variants near FRK/COL10A1 and VEGFA are associated with advanced age-related macular degeneration. Hum Mol Genet 2011;20:3699-709.

**Supplementary Notes**

**Genotyping and imputation**

Genotyping was performed using Illumina BeadChip; 974 individuals were genotyped using both OmniExpress and HumanExome, 574 individuals using HumanOmni2.5-8, and 28 individuals using OmniExpress. After a stringent quality control (QC), including Hardy-Weinberg equilibrium, P ≥ 1.0×10^-6^, minor allele frequency (MAF) ≥ 1%, and genotype call rate ≥ 95% (per SNP and per individual), we imputed them with a reference to 1,000 genomes’ project cosmopolitan haplotypes (1,092 samples from all over the world; available at IMPUTE2 website [https://mathgen.stats.ox.ac.uk/impute/impute_v2.html]; released at December 2013) in order to produce constant genotype data across each platform. QC was performed using PLINK ver1.07 (http://pngu.mgh.harvard.edu/~purcell/plink/), while imputation was performed using SHAPEIT2 (http://www.shapeit.fr/) and IMPUTE2 (https://mathgen.stats.ox.ac.uk/impute/impute_v2.html). Imputation status and quality are as follows.
